# Phenolic compounds as natural feed additives in poultry and swine diets: a review

**DOI:** 10.1186/s40104-021-00565-3

**Published:** 2021-04-07

**Authors:** Shad Mahfuz, Qinghui Shang, Xiangshu Piao

**Affiliations:** State Key Laboratory of Animal Nutrition, College of Animal Science and Technology, China Agricultural University, Beijing, 100193 China

**Keywords:** Antioxidant role, Immune response, Phenolic compounds, Production performance, Poultry, Swine

## Abstract

Due to ban on using antibiotics in feed industry, awareness of using natural feed additives have led to a great demand. The interest of plants phenolic compounds as a potential natural antioxidant source has been considered in research community due to their predictable potential role as feed additives in poultry and swine production. However, the mode of action for their functional role and dosage recommendation in animal diets are still remain indistinct. Taking into account, the present review study highlights an outline about the mode of action of phenolic compound and their experimental uses in poultry and swine focusing on the growth performance, antioxidant function, immune function, antimicrobial role and overall health status, justified with the past findings till to date. Finally, the present review study concluded that supplementation of phenolic compounds as natural feed additives may have a role on the antioxidant, immunity, antimicrobial and overall production performance in poultry and swine.

## Background

At present, the consumer’s demand for safe animal food is increasing which created a challenge for animal scientists and thus the interest for using natural feed additives has been articulated [1]. Phenolics are products of plant secondary metabolism. Phenolic compounds can be defined as any compound having a benzene ring with one (phenol) or more (polyphenol) hydroxyl group as esters, methylesters etc [1, 2]. A basic feature of phenolics are their significant antioxidant activities. In addition, some phenolics may have additional beneficial properties such as immunity, anti-inflammatory, gut health, and antimicrobial activity. Phenolic compounds have involved a great deal of research attention in nutrition and medicine of human during the last few decades. Phenolic compounds have highly antioxidant capacity that can play a vital role on health benefits [3]. However, the animal scientists are still recognizing phenolics as effective feed additives. With the exception of some members of the phenolics family (tannins, saponons, toxic alkaloids) the most of the plant extract did not show anti-nutritive in animal diets [4]. For example, high concentration of condensed tannins from certain plant extract in animal feed can precipitate protein digestion showed lower performance with nutrient retention in monogastric animals [5, 6]; whereas higher body weight with improve health status in chickens offered hydrolysable tannins was recorded [7]. This is because hydrolysable tannic acid extracted from wood was enrich with polyphenolic compounds [8, 9]. Dietary inclusion of polyphenols could enhance production performance and oxidative stability of food originated from farm animals [10]. Phenolic compounds as natural metabolites have been reported to counter oxidative stress, whereas such stress are correlated with severe metabolic disorders by having damage to cellular and extracellular macromolecules [11, 12]. Oxidative stress has very adverse effects on farm animal production. For example, it falls body weight by modifying optimum metabolism and reduces quality of meat by higher accumulating corticosterone in plasma that was accountable with paler color of breast meal in broiler chickens [13]. Furthermore, it could induce biological damage for DNA, protein, lipid, associated with various health effects that negatively alter production capacity in farm animals [14].

On the other hand, for more than seven decades, antibiotics have been applied at low levels for therapy and to promote growth performance in pigs and poultry [15]. Thus the antibiotics are commonly known as growth promoters for poultry and swine. It is true that antibiotics growth promoter could improve performance and could decrease mortality in pigs and poultry. But due to continue and excess uses of antibiotics in food for animal production has developed bacterial resistance to antibiotic growth promoters (AGPs) and created public health threats [15, 16]. In Europe, AGPs in animal feed have been banned since 2006, and currently most of developed countries have prohibited on using antibiotics in feed [15, 17]. Focusing on the beneficial roles of phenolics, it can be used as an effective natural feed additive in animals. Besides, agricultural by-products represent an excellent source of phenolic and antioxidant compounds that can be used as functional ingredients in livestock feeding [18].

Therefore, the goal of this review study was to highlight the uses and feasibility of phenolic compounds as potential natural feed additives in poultry and swine diets.

### Classification of phenolic compounds

Phenolic compounds can be a simple phenolic molecule or highly polymerized compounds. Those are found naturally as chain of mono and polysaccharides, with one or more phenolic groups and may connect to esters and methyl esters [12]. Wide-ranging phenolic compounds are found in nature for their diverse structure. Currently, about 8000 phenolic compounds structures have to be identified [12]. Common known phenolics includes phenolic acid, flavonoids, tannins, avenanthramides, alkylresorcinols, oligomeric proanthocyanidins and lignans etc [1, 2]. The source of phenolic compounds is mostly in plant tissues like grains, vegetables, fruits, trees and their extract [2, 19]. However, in human food, the common phenolic compounds are phenolic acids, flavonoids, and tannins [12]. Classification and structure of some common of phenolics were shown in Table 1 and Table 2 respectively.

**Table 1:** Classification of phenolic compounds

Items	Example	Source	Ref.
Soluble component	Phenolic acid: (gallic, protocatechuic, rosmarinic, gentisic, salicylic,vanillic, syringic, ferulic, caffeic, sinapic, cinnamic); flavonoid (flavones, flavonols, catechin, quercetin); quinines, phenolic diterpenes (carnasol, carnosic acid) and hydrolysable tannins	Millet, oat, sorghum, barley, wheat, rye, vegetable, fruits spices, herbs and their byproducts	[20–22]
Non-soluble component	Condensed tannins and lignins	Chestnut wood, pinewood and different trees and their byproducts	[9, 23, 24]
Others (aromatic compound)	Alcohols (borneal); aldehyde (cinnamaldehyde); ketons (carvones)	Aromatic plants and herbs and their byproducts, cinnamon, etc	[25, 26]

**Table 2:** Structure of phenolic compounds in plants

Class	Structure
Simple phenolics, benzoquinones	C6
Hydroxybenzoic acids	C6-C1
Acetophenones, phenylacetic acids	C6-C2
Hydroxycinnamic acids, phenylpropanoids	C6-C3
Xanthones	C6-C1-C6
Stilbenes, anthraquinones	C6-C2-C6
Flavonoids, isoflavonoids	C6-C3-C6
Lignans, neolignans	(C6-C3)_2_
Lignins	(C6-C3)_*n*_
Condensed tannins (proanthocyanidins or flavolans)	(C6-C3-C6)_*n*_

**Source:** Ref. [12]

### Application of phenolic compounds in poultry and swine diets

The perception of applying natural polyphenols in poultry and swine diets has been tested since 1989 [27] and later on it was considered for further research during the last two decades due to their potential biological roles [6, 28]. The biological role of phenolic compounds with their mode of action in farm animals were described in details below.

#### Phenolic compounds as growth promoters

The dietary uses of different plants and their extract as phenolic sources has examined as potential tools on improving growth performance, and decline mortality in farm animals [1]. The mechanism of phenolic compounds that may act as growth promoters for the farm animals, by enhancing digestive enzyme secretions (endogenous digestive enzymes, saliva, bile and mucus) and by decreasing the pathogenic bacterial number in GIT or by modulating gut morphology due to their antioxidant and anti-inflammatory functions [29, 30]. In addition, phenolic compounds originated from aromatic plants may improve the flavor and palatability of the feed and thus increase the feed intake and growth performance [1]. Scientific studies also noted that the higher growth rate and improve FCR in poultry fed with phenolic compounds were due to altering intestinal surface area as well as digestive enzyme activities which resulted in better nutrient absorption [31, 32]. Conversely, lower feed intake was also recorded at higher inclusion level of phenolic compounds at approximate above 1500 mg/kg diets in swine due to strong odor [33]. Thus it was recommended to avoid higher dosage in porcine diets due to their strong sensitive palate [34]. Phenolic compounds derived from aromatic plants and extracts had a role on keeping the optimum balance between the useful bacteria and pathogenic bacteria in GIT which help to maintaining sound gut health and ultimate growth enhancer [35–37]. The growth promoting role of phenolic compounds can also be justified by improving feed status while adding phenolic in diets leads better fermentation of diets resulting in more nutrient absorption and direct or indirect anabolic roles on host tissues [30, 38]. However, the mechanism of action of phenolic compounds may vary on its structure, inclusion levels, pharmacokinetics, experimental animal species, stage of animals and duration of trail [30]. Abdel-Wareth et al. [39] has conducted an experiment to evaluate the role of thyme and oregano essential oil (thymol and carvacrol) on broilers and observed higher average daily gain (ADG) with improved feed conversion ratio (FCR) in supplemented diets than control. The authors finally concluded that, thyme and oregano at the level of 15 or 20 g/kg diet can be applied as feed additives to improve growth performance in broilers. In addition, supplementation of oregano powder as phenolic compound at 150 mg/kg could increase about 8.4% higher ADG, along with 7.9% higher feed intake (FI) at the whole experimental period of broilers [40]. Furthermore, broilers supplemented with a mixture of thymol and carvacrol could enhance ADG with FCR via improving digestive enzyme activities [41]. Thyme oil at 1.0 g/kg diet was every effective as potential growth promoter in broilers reared under hot climate [42]. Compared with control diets, lower FCR in broilers fed with grape pomace concentrate at 60 g/kg was noted [32]. Similarly, higher body weight gain, final body weight with lower FCR were recorded in broilers offered with grape seed as a source of phenolic compounds [43]. Broilers fed with essential oil (thyme) at 0.3 g/kg was very useful to improve gut morphology and to increase digestive enzyme [44]. In addition, dietary inclusion of 0.3 g/kg cinnamon oil could enhance 5.1% daily weight gain and could improve nutrient digestibility while decrease nitrogen content in excreta of commercial broilers [45]. Dietary supplementation of essential oils derived from thyme (*Thymus vulgaris*), peppermint (*Mentha piperita*) and eucalyptus (*Eucalyptus globules*) at 150 ppm into drinking water could progress body weight, immune response, ileal structure and microflora in broilers [46]. Oral application of carvacol essential oil in broilers at 300 or 400 μL had a good role on body growth and intestinal barrier function via increasing the gene expression of occludin, claudin-1, claudin-5, ZO-1 and ZO-2 in mucosa [47]. In contrast, broilers fed with tannic acid as phenolic compound derived from chestnut wood at 2 g/kg had no effects on growth performance but could reduce the rate of footpad dermatitis lesions [48]. The antigrowth of broilers may due to the anti-nutritional effects of tannins that could attribute the protein binding capacity as well as could reduce the nutrient digestibility in birds at higher dosages [48]. Laying hen fed with oregano essential oil at 100 mg/kg could increase 5% egg production percent, and could improve egg weight, FCR, amylase and trypsin enzyme activities [49]. Higher egg production with lower FCR in laying hens fed with thyme and rosemary dry leaf powder as phenolic compounds at 0.9% level was also reported [50]. In contrast, dietary inclusion of thyme essential oil at 300 mg/kg could not improve egg production and egg quality in laying hen which may due to different inclusion level and form of supplement in diets of birds [51]. In Pekin ducks, a study noted that dietary inclusion of grape seed extract (anthocyanidins, catechins) as phenolic compounds at 0.2% diets could increase about 3.1% final body weight, and could improve FCR, antioxidant status, immunity, meat quality, and beneficial microflora of gut [52]. In another recent study, dietary supplementation with combination of essential oil (cinnamaldehyde 15%, and thymol 5%) with organic acids could enhance about 13.5% average daily weigh gain and about 5.6% higher final body weigh in weaned piglets [53]. However, supplementation of chestnut tannin (750 mg tannic acid/kg DM) as phenolic compounds at 0.3% level in Italian heavy pigs could not improve any performance parameters, slaughter traits and energy or protein utilization [54].

#### Phenolic compounds on meat quality

Phenolic compounds have direct or indirect role on meat quality. In a study by Luna et al. [55] used 150 mg/kg of thymol, or 150 mg/kg of carvacrol as natural polyphenol on lipid oxidation in broiler’s meat and observed lower values of TBARS in thigh muscle after 5 to 10 d of storage than the control group. The author concluded that thymol or carvacrol as natural antioxidant could be useful to improve meat quality of broilers. In contrast, supplementation of grape seed had no effects on physical and chemical composition of meat in broilers [43]. It has been reported that dietary supplementation of hesperidin could improve the oxidative stability via lowering the MDA levels in yolk of fresh eggs and eggs of 30 to 90 d of storages [56]. In a consequence study, supplementation of broiler diets with hesperidin and naringin as flavonoid compounds derived from citrus plants could decrease MDA levels in breast muscle after 6 d of storage but the supplementation diets had no effects on meat color, pH, and cooling loss percent in breast muscle of broilers [57]. In contrast, a significant higher value in meat color (lightness value) with higher pH, and water holding capacity in broilers fed with hesperidin and genistein at 20 mg/kg diets was reported [58]. Similarly, a lower MDA value in breast muscle after 15 d of storage was also recorded in broilers fed with hesperidin and genistein at 20 mg/kg diets [58]. The values for redness, saturation index, shear force, odor, taste, and overall sensory acceptability of breast meat were higher in broilers fed with oregano essential oils (*Poliomintha longiflora) *at 400 mg/L drinking water [59]. The author finally concluded that oregano essential oils can be used as natural additives to improve meat quality of broilers. Gallic acid and linoleic acid at 1% level also could improve antioxidant capacity and the content of phenolic was about 4% higher in breast muscle of experimental broilers [60]. Thyme essential oil has been proved to inactivate the *Salmonella enteritidis* on ready to eat turkey meat products during preservation [61]. Microencapsulated complex of organic acids and essential oils (thymol 1.7% and vanillin 1.0%) at 0.2% level could improve red color value of meat and decreased drip loss about 30% percent in weaning to finishing pigs [62]. In addition, oregano (*Origanum vulgare*) essential oil at 0.2% level could improve the oxidative stability of meat in pigs [63].

#### Phenolic compounds on antioxidant function

Phenols have naturally antioxidants properties, and can protect biomolecules (proteins, nucleic acids, polyunsaturated lipids, and sugars) from oxidative damage via free radical-mediated reactions [64]. The basic mode of action for anti-oxidative power of phenolic compounds are related with reducing properties as hydrogen or electron donation that made those compounds as free-radical scavengers (antioxidants). Further, those compounds have metal chelating activities, especially for iron and copper, and can suppress the formation of metal-catalyzed free radicals [12]. Those anti-oxidative role depends on hydroxyl group number, position and the relationship with the carboxyl functional groups [12, 65]. Moreover, the antioxidant properties of phenolic compounds are associated with the structural function relationship [66], glycosylation, and the atoms in the rings [67]. The another pathway for the anti-oxidative role of phenolic compounds may behinds on decreasing the DPPH with an effective concentration more than 50% and to reduce lipid oxidative reaction with an inhibitory concentration more than 50% (IC_50_) [15]. In brief, the donating capacity of hydrogens or electrons and to delocalizing the unpaired electrons within phenolic ring are the main mechanism of protecting biological molecules against oxidation [37, 15]. Reactive oxygen radicals can attack the surface of the intestinal mucosa, and prevent the absorption of nutrients whereas antioxidant plays a vital role on neutralizing reactive oxygen radicals and keeps better environment in intestinal surface [30].

Broiler diets supplemented with a mixture of thymol and carvacrol could increase SOD, GSH-Px, and reduced MDA levels in thigh muscle, serum and liver sample [41]. The oxidative susceptibility of liver and breast muscle was improved in broilers fed with thymol (200 mg/kg); tannic acid and gallic acid (5 g/kg) diets [10]. In a study, broilers offered grape seed as a source of total phenol 55.5 g/kg, total flavonoids19.5 g/kg, and total tannin 9.4 g/kg diets [43]. The study observed higher SOD, CAT, GSH-Px, GST, GSH value and lower TBARS value in plasma of broilers offered grape seed as polyphenolic compounds [43]. The author justified that the polyphenol present in grape seed has been absorbed sufficiently to enhance the antioxidant function in experimental broilers. Similarly, dietary vitamin E could be replaced with grape polyphenol at 75 mg/kg on similar antioxidant status and immune response in broilers [68]. The polyphenolic compounds exhibit in tea have been widely applied as antioxidants in animal production as well as to prevent diseases [69]. In a broilers trail the basal diets were contaminated with ochratoxin as stress factor whereas phenolic product was applied as dietary supplement and observed the higher antioxidant status in blood, and higher concentrations of non-enzymatic antioxidants in the liver and breast muscles [70]. Boilers fed with essential oil (carvacrol, cinnamaldehyde and capsicum oleoresin) at 100 mg/kg could improve the hepatic antioxidant including carotene, coenzyme and total vitamin E [71]. In addition, 150 mg/kg of oregano powder as source of phenolic compounds was used in broiler diets and found higher T-AOC with lower MDA value in serum of experimental broilers [40]. Moreover, essential oil derived from herb rosemary at 20 mL/100 kg could improve antioxidant status via increasing activity of glutathione peroxidase in heat-stressed laying hens [72]. Lower serum MDA level in laying hens fed with thyme and rosemary dry leaf powder as source of phenolic compounds at 0.9% level was also reported [50]. The replacement of 50% dietary vitamin E with polyphenols did not alter the growth performance but could improve the antioxidant status of sows or their offspring [73]. In piglets, dietary supplementation of polyphenol mixture originated from apples, grape seeds, green tea leaves and olive leaves could reduce plasma MDA value [74]. In a recent study, diets supplemented with grape seed procyanidins as phenolic compounds at 40 mg/kg could successfully enhance the resistance to weanling stress via increasing the expression of GSH-Px, SOD and CAT genes related with antioxidant in liver and could decrease MDA level in serum, liver and muscle tissue [75].

#### Phenolic compounds on immune function

Phenolic compounds originated from various aromatic plants have been proved as immune enhancing role. The anti-inflammatory function of phenolic compounds has become great interest due to their suppressive role of inflammatory prostaglandins and nitric oxide production that could decrease inflammatory process [30]. Phenolic compounds possess anti-inflammatory properties which is one of the major aim to use phenolic compounds as feed additives in farm animal’s diets [76]. The basic mode of action of phenolic compounds on immune function is to produce immunoglobulin and secretion of cytokine, increase phagocytosis, and bolstering to release of interferon-ү [1]. Polyphenols could active mononuclear cells and could increase phagocytic response by influencing MAPK and nuclear factor κB (NF-κB) signaling pathways [76]. The phenolic compounds enhanced the duodenal function and nutrient absorption which promote higher immune status and ultimate growth in broilers [77, 78]. Dietary polyphenols could improve the gut health and immunity of monogastric animals by stimulating immunoglobulins as well as hindering the secretion of pro-inflammatory cytokines [79]. In addition, the phenolic compounds can play over-expression of antioxidant enzymes, that might down-regulate the inflammation [80]. Different flavonoids and terpenoids have noted to own anti-inflammatory properties [81]. The anti-inflammatory properties of essential oils have been reported for their interaction with signaling cascades involving various cytokines and regulatory transcription factors as well as for their pro-inflammatory gene expression capacities [76, 82]. Similarly, different alkaloids (isoquinoline, acetylsalicylic acid) have also been proved as anti-inflammatory role because those could decrease the expression of pro-inflammatory cytokines [76, 83]. Those alkaloids enriched with phenolic compounds can play a role on gut health by affecting the inflammation cascade with the inhibition of the NF-kB activation [76]. Higher cellular and humoral immune response was found in broilers fed with essential oil of thymol and carvacrol [41, 84]. Plant flavonoids genistein (5 mg/kg) and hesperidin (20 mg/kg) have applied in lipopolysaccharide (LPS) challenged broilers and showed to be effective role on immune-stimulation and improved gut morphology [85]. Moreover, the immunomodulatory function of plant polyphenol has been reported via dropping the expression of interleukins IL-4, IL-13, IL-18 and IFN-γ in LPS challenged broilers fed with phenolic rich diets [86]. Dietary supplementation of saponins, phenolic compounds originated from soapnut (*Sapindus mukorossi*) at 150 mg/kg could improve the cell-mediated and humoral immune response of broilers without adverse effects on optimum body weight [87]. Inclusion of thyme essential oil at 3 g/kg could improve serum IgG, IgA, antibody titer against ND vaccine in broilers and could eliminate the adverse effects of ochratoxin A and aflatoxin B_1_ in broiler diets [88]. In broilers challenged with LPS, dietary supplementation of carvacrol essential oils as phenolic compound at 200 μL/L could prevent the expression of inflammatory cytokines that ensured the anti-inflammatory role of carvacrol in broilers [89]. In a recent study, resveratrol a phenolic compound at 500 mg/kg could regulate the immune function and inflammatory response, by inhibiting different signaling pathways like NF-κB, MAPK, and PI3K/AKT caused by heat stress in yellow feather broilers [90]. In addition, higher expression of T helper 1 cytokine (interferon-γ) and lower expression of T helper 2 cytokine (IL-6) were noted in laying hens fed with proanthocyanidin-rich extract from *Pinus radiata* bark (20 mg/kg) [91]. The author finally stated that proanthocyanidin had an immunomodulatory effect in chickens. Higher monocyte counts in hens fed with fennel essential oil at 300 mg/kg [92] and increased number of lymphocyte with thyme powder at 0.2% were reported as good health indicators in laying hens [93]. Due to the extended production life and long vaccination schedules of the laying hens from day-old to curled age may have an indirect role on supporting the immune function with phenolic compounds in diets [94]. Laying hens fed with mixed essential oil originated from *Thymus vulgaris, Mentha piperita, Rosmarinus offisinalis* and *Anethum graveolens* at 200 mg/kg could improve antibody response against ND vaccine and SRBC [95].

Piglets fed with polyphenolic rich diets showed lower expression of different pro-inflammatory genes in duodenum, ileum and colon than the control diets [96]. In addition, dietary supplementation of phenolic rich soy isoflavones showed improve immune function with lower diarrhea rate, and lower concentrations of endotoxin in plasma of piglets challenged with LPS [97]. A significantly lower inflammatory mediators NF-κB and Nrf2 in duodenal mucosa resulted in lower risk of intestinal diseases was also reported in pigs fed with phenolic rich supplemented diets containing grape seed and grape pomace extract [98]. In a trail for piglets subjected to oxidative stress (diquat injection, intraperitoneal) showed tea polyphenols could influence the activities of T lymphocyte, increased the ratio of CD4^+^/CD8^+^ ensured the recovery activities of immune damages caused by oxidative stress. Further it could enhance the cell-mediated immune response and reduce the secretion of pro-inflammatory cytokines such as IFN-γ, which ensured the immunomodulatory role of tea polyphenols [99]. The mechanism for selective inhibition of IFN-γ signaling, by downregulating STAT_1_ activation and T-bet expression of CD4 (+) T cell in colonic lamina propria. In a recent study, dietary supplementation of tea polyphenol at 100 mg/kg in piglets could improve the intestinal mucosal immunity via increasing the content of IL-2, IL-10 in jejunum and ileum as well as activated the Notch2 signaling pathway in small intestine [100].

#### Phenolic compounds on lipid metabolism

The anticholesteremic effects of phenolic compounds has been proved in different *in vivo* trails. For example, Park et al. [101] has applied rutin and tannic acid as phenolic compounds in rats to evaluate the lipid metabolism and observed that both phenolic compounds could significantly reduce the cholesterol level in plasma and liver. It was hypothesized that supplemented phenolic compounds stimulated the excretion of fecal sterols, and reduced absorption of dietary cholesterol which resulted lower plasma and hepatic cholesterol. Similarly, supplementation of phenolic compounds as feed additives in farm animal diets has shown positive effects against lipid oxidation [30]. Lower plasma total cholesterol (TC), LDL-C were reported in broilers fed with thyme (*Thymus vulgaris*) extract at 0.2% to 0.6% diets and the decrease rate were 36% to 40% for TC and 63% to 70% for LDL-C [102]. Broilers diets supplemented with thymol; tannic acid and gallic acid showed about 12% lower TC in liver and higher PUFA in breast muscle [10]. In addition, about 10% lower total lipid, triglycerides and about 22% lower cholesterol were found in broilers offered polyphenol rich grape seed at 10 to 40 g/kg diets [43]. The bioflavonoids (genistein and hesperidin) could improve the fatty acid composition and lipid metabolite in broilers [103]. In breeder quail, fed with dill essential oil (*Anethum graveolens* L.) at 250 mg/kg diets could reduce about 21.52% total cholesterol (TC) in serum [104]. The mode of action of polyphenol on cholesterol reduction is that it contains multiple phenolic compounds which inhibit the oxidation of cholesterol resulted lower deposition of lipids in blood vessels. Furthermore, it prevents the oxidation of unsaturated fatty acids resulted lower deposition of cholesterol in serum and maintenance a good balance of normal entry and exit of lipids in blood vessel and thus have an anti-cholesteremic effects [69].

#### Phenolic compounds on antimicrobial function

The antimicrobial role of pohenolic compounds originated from aromatic plants and their derivatives have been described by several scientists [105, 106]. Phenolic compounds showed inhibitory activities against Gram negative and Gram positive bacteria [1]. The mode of action of phenolic compounds behinds their lipophilic nature which can amass in lipid bilayer of bacterial cell membrane and mitochondria, resulted in distracting regular functions [1]. In addition, those compounds could increase the permeability of inner bacterial membrane, decrease ATP production and inhibit the DNA gyrase that involves the mechanism of DNA and RNA synthesis of bacteria. Moreover, phenolic causes cell homeostasis and resulted in cell death by losing ions as the denaturation of cellular protein is responsible for bacterial cell death [105, 107]. Besides, the antimicrobial role of phenolic compounds is due to their structure. The hydroxyl (—OH) groups in phenolic compounds exhibit bactericidal activities [101].

It has been described that dietary supplementation of plant extract or herbs, could effective in counter to intestinal colonization of *E. coli* and *Clostridium perfringens* due to antimicrobial properties of existing phenolic compounds (carvacrol and thymol) [108, 109]. In addition, broilers offered with phytogenic feed additives could attenuate the pathogenic effects of *Eimeria* infection [110, 111]. Moreover, essential oil (oregano oil) originated from different plants have shown effective against gut parasites (*Cryptosporidium* spp.) [112]. Dandelion herb has been reported to have antibacterial role especially against *Staphylococcus aureus* and *E. coli* in poultry [113]. Similarly, broilers diets supplemented with phenolic rich grape concentrate or seed extract could alter intestinal morphology and microbial population [32]. In their studies, higher number of *Lactobacillus* and *Enterococcus* spp. in ileal content with lower number of *Clostridium* was found in broilers. In contrast, broiler chickens supplemented with thyme and oregano could decrease *Lactobacillus* counts in crop, and small intestine, respectively [39]. However, no significant differences for *Lactobacillus* count in cecum was noted. The authors justified that the supplementation dosages up to 30 g/kg was not enough to alter the gut community in their studies. Decreased number of *E. coli* in broilers fed with grape seed extract was also recorded [114]. In a recent study, broilers fed with essential oil (thymol) along with organic acid at 0.3 g/kg diet could decrease the number of *E. coli* in ileal digesta [115]. The authors justified that the decreased number of *E. coli* could enhance the absorptive capacity of intestine via improving the epithelial cells to regenerate villus area of host. The antimicrobial properties of thyme essential oil against *Salmonella* spp.; and oregano essential oil against total viable bacteria and *Pseudomonas* spp. on turkey products have been reported respectively [61, 116]. In very recent study reported that *Campylobacter jejuni* of chicken can contaminate carcass at slaughter which is responsible for gastroenteritis in humans. However, the colonization effects *C. jejuni* was successfully reduced in broilers fed with carvacrol a phenolic compounds originated from oregano oil (*Origanum glandulosum*) [117]. A similar finding against *Campylobacter* colonization in broilers fed with combination of thymol, and carvacrol at 0.5% level was also recorded [118]. In addition, lower number of *Streptococcus* spp., *Escherichia coli* and higher number of *Lactobacillus* spp. in ileum of broilers fed with grape seed was recorded [43].

Oral administration of 300 or 400 μL carvacrol essential oils could reduce *Salmonella* spp. and *Escherichia coli* in gut of broilers [47]. Furthermore, the lower number of *Clostridium* was found in ileum of broilers fed with essential oil (thyme 1.7% and vanillin 1%) at 5 g/kg dietary supplementation [119]. In addition, thyme essential oil with benzoic acid at 800 g/t diets had bacteriostatic role on *Salmonella enteritidis* in broilers [120]. Several authors justified that the polyphenolic compounds could enhance bactericidal activities which prevent growth of pathogenic bacteria in intestine where as some beneficial bacteria like *Lactobacillus* spp*.* and *Bifidobacterium* spp. could play a role on metabolism of phenolic compounds that provides energy to cells [32, 121, 122]. Higher number of enteric *Bifidobacterium *and* Lactobacillus* spp. whereas the lower number of *Escherichia coli* and *Salmonella* were noted in laying hens fed with oregano essential oil at 100 mg/kg [49]. Laying hens diets supplemented with essential oil (thyme oil) in combination of organic acid at 300 mg/kg could increase the number of *Bifidobacterium* spp. in cecal digesta [123]. Similarly, lower number of enteric *Escherichia coli* was found in laying hen fed thyme at 0.5% level of supplementation [124]. However, feeding molded laying hen with oregano essential oil at 24 mg/kg had no any effects on enteric bacterial count which may due to higher aged hens failed to metabolize the active ingredients of test diets [125]. Piglets fed with commercial mixture of phytochemicals showed lower number of fecal *Salmonella* and *E. coli* [126] and higher number of *Lactobacillus* spp. with benzoic acid and thyme supplementation [127]. Furthermore, combination of chestnut wood tannins and organic acids could decrease the harmful *E. coli* and increase the beneficial lactic acid bacteria in piglets during 82 to 127 d of evaluation [23]. Similarly, piglets fed with polyphenolic rich (grape extract or hop) supplemented diets showed lower number of *Clostridium *cluster in the faecal microbiota [96]. In a pathogenic challenge trail where piglets were infected with enterotoxigenic *E. coli* offered commercial polyphenol rich diets and the lower diarrhea rate was recorded [128].

The application of phenolic compounds with major physiological responses in poultry and swine were presented in Table 3 and Table 4 respectively.

**Table 3:** Application of phenolic compounds with major physiological responses in poultry

Phenolic compounds	Study design	Main findings	Reference
***Broilers***			
Polyphenol	Type: Ross 308-male dose: 0, 20, 40, 60 g/kg source: grape pomace (*Vitis vinifera)* form: powder duration: 0-24 days	●no effects on FI, ADG, and FCR ●higher thigh and drumstick weight ●lower abdominal fat weight ●lower TG, TC, LDL, AST, MDA in plasma ●higher SOD, GSH-Px in plasma ●higher antibody response in plasma	[129]
Hydrolysable tannic acid	Type: Arbor Acre male form: powder dose: 1000 mg/kg source: commercial (chestnut wood extract-75% tannin) duration: 0-42 days	●higher ADG, final body weight and improve FCR ●higher CP retention ●pH at 24 h was higher in breast muscle ●higher T-AOC, GSH-Px, SOD in serum, breast and thigh muscle ●lower serum TC, LDL-C and urea N	[22]
Essential oil	Type: Ross 308 dose: 0.4 mL/L source: dry herb (lavender flowers) form: liquid duration: 0-42 days	●higher ADG and final body weight ●improved FCR ●no effects on TC, TG, glucose, and uric acid concentration in serum ●lower *Escherichia coli* and coliform in ileum ●higher probiotic bacteria in ileum	[130]
Polyphenol (resveratrol)	Type: Yellow feather broilers dose: 200, 350 and 500 mg/kg source: dry herb (*Polygonum cuspidatum*) form: powder duration: 28-42 days	●higher ADG ●higher T-SOD, CAT, GSH in serum ●lower corticosterone, adrenocorticotropic hormone ●lower TC, TG, uric acid, AST, ALT and MDA in serum	[131]
Polyphenols (resveratrol)	Type: Arbor Acres male broilers dose: 300 and 600 mg/kg source: commercial form: powder duration: 0-42 days	●higher body weight gain ●improve FCR ●lower ALT, and AST in serum ●higher IgG, IgM, SOD in serum ●lower *E. coli* in cecum ●higher ratio of villus height to crypt depth of duodenum	[132]
Essential oil (cinnamaldehyde)	Type: Vencobb-400 broilers dose: 0.3 g/kg source: cinnamon bark oil form: extract duration: 0-42 days	●higher villi height of duodenum, jejunum and ileum ●lower number of *Escherichia coli* in pre-caecal contents ●higher antibody titers against NDV ●lower TC in serum ●higher SOD in serum ●no effects on TP, glucose and TG in serum	[133]
Polyphenols	Type: Cobb male broilers dose: 5, 7.5 and 10 g/kg source: grape pomace (*Vitis vinifera*) form: powder duration: 3-28 days	●no effects on FI, ADG, and FCR ●no effects on DM, CP, EE, and GE retention ●no effects on inner organs weight ●decreased TC in serum ●higher IgG in serum ●decreased thiobarbituric acid reactive substances values of breast meat ●lower redness value of meat during storage	[134]
Polyphenols (resveratrol)	Type: Cobb male broilers dose: 0 and 400 mg/kg source: commercial form: powder duration: 0-21 days	●higher T-AOC, CAT and lower MDA in breast muscle ●lower lightness value, and drip loss of meat ●higher redness value and pH-24h of meat	[135]
Condensed tannins	Type: Ross 308 dose: 125, 250, 500, 1000 and 2000 ppm source: grape seed extract (total polyphenol > 40%) form: powder duration: 0-42 days	●no effects on growth performance and mortality ●lower TC and LDL-C in serum ●higher antibody titer against NDV ●decrease MDA content in muscle tissue	[136]
Polyphenols (proanthocyanidins)	Type: Cobb-500 dose: 7.5, 15 and 30 mg/kg source: commercial (grape proanthocyanidins) form: powder duration: 0-42 days	●no effects on FI, ADG, and FCR ●improved jejunum morphology ●higher T-SOD, ALT, ALP, and CRE concentration in serum ●lower MDA value in serum	[137]
Polyphenols (flavonoids, catechins, and epicatechins)	Type: Hubbard broilers dose: 25, 50 and 75 ppm in replace of vitamin E form: powder source: grape seed extract duration: 0-35 days	●higher total phenolic contents in breast and leg muscles ●higher NDV and IBDV antibody titers ●lower thiobarbituric acid reactive substances in breast and leg muscles	[68]
Polyphenols	Type: Cobb male broilers dose: 0, 5% and 10% source: commercial (grape pomace) form: powder duration: 0-21 days	●no effects ADG, FI and FCR ●higher oxidative stability and PUFA content in thigh muscle ●lower SFA content in thigh muscle	[138]
Thyme oil	Type: Ross 308 broilers dose: 0.5 g/kg source: commercial (*T. vulgaris*) form: powder duration: 0-35 days	●lower MDA in duodenal mucosa and kidney ●higher IgA in duodenal mucosa ●improve intestinal barrier integrity	[139]
Flavonoids (genistein and hesperidin)	Type: Arbor Acre broilers dose: 5 mg/kg and 20 mg/kg and mixture (1:4) source: commercial form: powder duration: 1-42 days	●higher T-AOC, SOD in serum ●lower MDA content in serum ●lower TC, LDL-C, TG in serum and breast muscle ●improved PUFA, and ration of n-6/n-3 fatty acid	[103]
Essential oil	Type: Arbor Acres dose: 120 ppm source: oregano, anis and citrus peel tree form: extract duration: 0-42 days	●lower serum cholesterol ●lower ammonia concentration in ileum ●higher total polyphenolic compounds and total flavonoids in serum	[140]
Essential oil (thymol and cinnamaldehyd)	Type: Ross broilers dose: 0.1 g/kg (15 g/t thymol and 5 g/t cinnamaldehyde) source: commercial blend oil form: powder duration: 0-42 days	●higher ADG ●higher number of *Lactobacillus* and *Escherichia coli* in cecum ●higher proportion of caecal butyrate ●decreased proportion of caecal caecal acetic acid and propionic acid	[141]
Tannic acid	Type: Ross 308 dose: 20 g/kg source: commercial product form: powder duration: 17-27 days	●lower body weight and FCR ● lower lactate dehydrogenase, aspartate aminotransferase and alanine aminotransferase in serum ●higher relative weight of intestine	[142]
***Laying hens***			
Essential oil (peppermint)	Type: Bovans Brown laying hens dose: 0, 74, 148, 222, and 296 mg/kg source: dry leaf extract (*Mentha piperita)* form: liquid duration: 32-44 weeks	●higher FI, egg production, egg weight, egg mass, eggshell thickness, and haugh unit ●higher CP, EE and P digestibility ●higher TP in serum ●lower serum cholesterol	[143]
Essential oil	Type: Hy-line Brown layer dose: 0, 200, 400 and 600 mg/kg source: star anise (*Illicium verum*) form: liquid duration: 29-31weeks	●higher CP, OM digestibility ●higher metabolic efficiency of lysine, methionine, arginine, and threonine ●no effects on DM, EE and GE retention	[144]
Thymol and carvacrol	Type: Bovans-White dose: 1000 mg/kg each source: *Thymbra spicata* and *Rosemarinus officinalis* extract form: dry leaf powder duration: 48-56 weeks	●lower TC and TG in serum ●lower egg production and egg weight ●no effects on FCR, egg shell thickness, yolk color and haugh unit	[145]
Polyphenols	Type: Yueqinhuang laying hens dose: 0, 0.5, 0.8 and 1.2 g/kg source: eucalyptus leaves form: extract powder duration: 35-44 weeks	●higher egg production, egg mass, egg shell thickness ●lower MDA and cholesterol in egg yolk ●higher GSH-Px, T-SOD, T-AOC in serum ●higher pH at 45 min with higher redness value of meat ●lower drip loss of meat	[146]
Polyphenol	Type: Hy-line Brown dose: 200 mg/kg source: commercial (tea polyphenol and tea catechins) form: powder duration: 65-74 weeks	●higher egg production ●improved FCR ●higher albumen height with haugh unit ●higher protein sulfhydryl content of the albumen ●lower protein carbonyl content and protein surface hydrophobicity of albumen	[147]
Essential oil	Type: Lohmann White form: powder source: commercial dose: 0, 50, 100 and 150 mg/kg duration: 54-65 weeks	●no effects on egg production, egg weight, egg quality, FI and FCR ●higher CP digestibility ●no effects on energy utilization ●no effects on SFA, MUFA and PUFA composition in egg yolk	[148]
Essential oil	Type: Hy-line layer form: fine granulates powder source: oregano dry herbs dose: 50, 100 and 150 mg/kg duration: 30-37 weeks	●no effects on egg shell weight, yolk index and haugh unit ●lower crypt depth and higher villus height with villus height to crypt depth ratio of duodenum ●higher gene expression on glucose transporter 2, peptide transporter 1, sodium-glucose cotransporter 1 in duodenum and jejunum	[49]
Essential oil mixture	Type: Hy-line White dose: 0, 100 and 200 mg/kg source: *Thymus vulgaris, Mentha piperita, Rosmarinus officinalis* and *Anethum graveolens* form: powder duration: 40-51 weeks	●lower TC, TG, AST, ALT, in serum ●lower MDA in serum and liver sample ●higher eggshell hardness and thickness	[149]
Essential oil (peppermint/thyme)	Type: Lohmann LSL-lite dose: 0 and 1000 mg/kg each source: dry herb extracts form: powder duration: 40-48 weeks	●higher egg production, egg mass ●lower FCR ●higher egg shell thickness, haugh unit ●lower serum cholesterol	[150]
Thyme and fennel	Type: Hy-Line White dose: 0 and 40 mg/kg each source: dry herbs (*Thymus vulgaris* and *Foeniculum vulgare*) form: alcoholic extracts duration: 26-38 weeks	●higher egg weight and egg mass ●higher egg yolk color ●lower egg yolk cholesterol ●improve egg yolk omega-3 fatty acid	[151]
Essential oil	Type: White leghorn (Lohman) dose: 24 mg/kg source: oregano dry herbs form: powder duration: 82-106 weeks	●higher egg production with lower FCR in forced molted hens ●higher SOD value and lower MDA value in liver sample	[125]
Thymol	Type: Hi-sex Brown source: *Thymus vulgaris* dry herb form: powder dose: 3, 6 and 9 g/kg duration: 36-52 weeks	●no effects on FI, ADG and FCR ●higher egg weight ●higher SOD, GSH-PX and lower MDA in serum ●higher IgG in serum ●lower LDL-C in serum	[152]
Essential oil	Type: Lohmann LSL-lite laying hens dose: 40 mg/kg source: cinnamon bark form: powder duration: 42-50 weeks	●higher egg production, egg weight and egg mass ●no effects on FI and body weight ●no effects on TC,TG, uric acid and albumin content in serum	[153]
Fennel (anethole, fenchone and estragole)	Type: White leghorn dose: 0, 10 and 20 g/kg source: fennel fruits (*Foeniculum vulgare)* form: dry fruit powder duration: 42-46 weeks	●lower serum MDA ●lower TC and TG in egg yolk	[154]
Polyphenols	Type: Hy-line White (Leghorn) dose: 0, 2.5, 5, 7.5 and 10 g/kg source: *Echinacea purpurea* form: dry leaf extract powder duration: 43-53 weeks	●higher egg production and egg mass ●lower TC, TG, in serum ●lower cholesterol in egg yolk ●higher HDL in serum	[155]
Peppermint/ menthol/ menthone/ isomenthone/ menthyl acetate/ cineol	Type: Hy-line Brown dose: 0, 5, 10, 15 and 20 g/kg source: dry herb (*Mentha piperita)* form: leaves powder duration: 64-76 weeks	●higher FI, egg production, egg weight, egg mass, eggshell thickness, haugh unit ●lower FCR ●lower serum cholesterol ●higher TP in serum	[156]
Essential oil mixture (carvacrol, thymol, 1:8-cineole, p-cymene and limonene)	Type: ATAK-S laying hens dose: 0, 3 and 6 mg/kg source: commercial form: powder duration: 52-68 weeks	●no effects on FI, egg production, egg weight and FCR ●no effects on glucose, TC and TG in serum ●no effects on antibody titers against NDV, IB and IBDV	[157]
Thyme	Type: Lohmann LSL-white dose: 0, 0.1%, 0.5% and 1% source: dry herbs (*Thymus vulgaris*) form: powder duration: 24-36 weeks	●higher egg production with lower FCR ●decrease concentration of *E. coli* in feces	[124]
***Other birds***			
Polyphenol (resveratrol)	Type: quail dose: 0, 200 and 400 mg/kg source: dry herbs (*Polygonum cuspidatum*) form: powder duration: 4-16 weeks	●no effects on FI, egg production ●no effects on vitamin A concentration in serum and egg yolk ●higher vitamin E in serum ●lower MDA in serum and egg yolk	[158]
Thymol	Type: quail (female) dose: 2 g/kg (80 mg/bird per day) source: commercial form: powder duration:100-130 days	●no effects on corticosterone concentrations in plasma ●higher albumen, glucose, globulins, TP in plasma ●higher inflammatory responses ●higher heterophil to lymphocyte ratio in blood	[159]
Essential oil	Type: quail breeder dose: 250 mg/kg source: dry herbs (*Trachyspermum ammi* and *Anethum graveolens*) form: powder duration: 8-18 weeks	●no effects on egg production ●improved FCR ●no effects on TG, glucose and HDL in serum ●lower TC in serum ●higher number of *Lactobacillius* in cecum ●no effects on *E. coli* number in cecum	[104]
Phenolic compounds (peppermint)	Type: quail dose: 10, 20, 30 and 40 g/kg source: dry herbs (*Mentha piperita)* form: powder duration: 7-35 days	●no effects of FI and ADG ●higher length of small intestine, villus height, villus width, crypt depth, and villus area ●higher lactic acid bacteria in ileum ●lower *Escherichia coli* in ileum ●no effects of inner organs weight	[160]
Polyphenols (saponins, flavonoids)	Type: Japanese quail dose: 0, 1%, 3% and 5% source: dry herbs (*Astragalus membranaceus*) form: powder duration: 0-35 days	●higher FI, and weight gain ●higher weight of thymus gland and bursa of fabricius ●higher IgA, C3, C4 in serum ●higher T-AOC, GSH-Px, CAT in serum ●higher *Lactobacillius* and *bifidobacterium* in intestine ●lower *Escherichia* number in intestine ●no effects on TP, TC, TG, AST, and ALT in serum	[161]
Thymol	Type: quail dose: 0, 2, 4 and 6.5 g/kg source: commercial form: powder duration: 85-128 days	●lower SFA in egg yolk ●higher PUFA in egg yolk ●no effects on body weight gain, FI, egg production, and egg weight	[162]
Polyphenols (anthocyanidins, catechins)	Type: duckling (Pekin- female) dose: 0, 0.1% and 0.2% source: grape seed extract form: powder duration: 0-6 weeks	●higher ADG, and final body weight with improved FCR ●higher SOD, GSH-Px, T-AOC, CAT, IgG, IL-2 and lower MDA in serum ●lower abdominal fat ●lower ileal *Escherichia coli* but higher ileal *Lactobacilli*	[52]
Essential oil (oregano oil)	Type: duckling (Cherry valley) dose: 150 and 300 mg/kg source: commercial-oregano (5% thymol, 65% carvacrol and 30% carrier) form: powder duration:11-42 days	●no effects on final body weight, ADG, FI, and FCR ●lower number of coliform bacterial in cecum ●no effects on TC, TG, AST, ALT, glucose, and TP in serum	[163]
Essential oil (oregano oil)	Type: duckling (Cherry valley) dose: 100 mg/kg source: commercial (oregano oil) form: powder duration:1-35 days	●no effects on ADG, FCR, DM, CP, EE, Ash and GE digestibility ●higher villus height to crypt depth ratio in jejunum ●higher SOD in serum and T-AOC in jejunum mucosa ●lower MDA in serum and liver tissue ●decreased mRNA expression of ZO-3 and sIgA	[164]
Thymol	Type: turkey poults dose: 30 mg/kg source: commercial extract form: powder duration:180-236 days	●improved FCR ●no effects on ADG, lactic acid bacteria and coliform count in crop, ileum and cecum ●lower MDA level in liver, thigh and breast muscle ●higher GSH-Px, GST activities in liver, thigh and breast muscle	[165]
Mixed essential oil	Type: turkey dose: 1 mL/L source: commercial extract form: liquid duration:175-178 days	●higher percent of CD4^+^ T lymphocytes in the thymus and the spleen ●higher percent of CD8^+^ T lymphocytes in the cecal tonsils and the blood ●higher percent of CD4^+^ CD8^+^ T lymphocytes in the thymus and ileal mucosa	[166]
Mixed essential oil	Type: turkey dose: 0.3 g/kg source: commercial form: powder duration: 0-140 days	●no effects on ADG, FCR and carcass weight ●lower pH of crop content ●higher α-glucosidase in the ileal digesta ●no effects on SCFA production in the caeca	[167]

**Table 4:** Application of phenolic compounds with major physiological responses in swine

Phenolic compounds	Study design	Main findings	Reference
Polyphenols (procyanidins)	Type: crossbred piglets (Duroc × Landrace × Yorkshine) dose: 50, 100 and 150 mg/kg source: commercial (grape seed procyanidins) form: powder duration: 1-28 days	●higher maltase and sucrase in jejunum mucosa ●lower urea nitrogen, diamine oxidase, endotoxin in blood ●lower digestibility of DM, GE, and CP ●decreased lipase and amylase activities of pancreas and duodenum	[168]
Polyphenols	Type: weaned piglets (Songliao black pigs) dose: 5% source: grape pomace form: powder duration: 28-56 days	●no effects on ADG, FI, and FCR ●higher number of *Lactobacillus delbrueckii, Olsenella umbonata* and *Selenomonas bovis* in caecum ●higher villus height and villus height/crypt depth ratio of jejunum ●lower MRNA expression of proinflammatory cytokines (IL-1β, IL-8, IL-6 and TNF-α) ●higher IgG in serum	[169]
Phenolic compounds	Type: piglets (Landrace × Large White × Duroc) dose: 9% solid source: grape pomace form: silage duration: 20-50 days	●higher ADG and final body weight ●higher GSH, H_2_O_2_ and TAC in intestinal tissue ●decreased in TBARS and CARB in intestinal tissue ●higher lactic acid bacteria and Bifidobacteria in feces ●lower Enterobacteriacae and *Campylobacter jejuni* in feces ●higher n-3 fatty acids in meat ●lower n-6/n-3 ratio in meat	[170]
Polyphenol	Type: male finishing pigs (Yorkshire × Duroc × Landrace) dose: 5% source: fermented apple pomace form: silage duration: 53 days	●no effects on ADG, and final body weight ●lower DM intake with higher FCR ●no effects on carcass weight, and back fat thickness ●higher moisture content with lower ash and water holding capacity of meat ●higher linoleic acid (C18:2n-6), linolenic acid (C18:3) and arachidic acid (C20:0) and total PUFA in back fat ●lower palmitic acid (C16:0), palmitoleic acid (C16:1) and heptadecenoic acid (C17:1)	[171]
Procyanidins	Type: piglets (Duroc × Landrace × Yorkshine) dose: 50, 100 and 150 mg/kg source: grape seed extract form: powder duration: 0-28 days	●no effects on body weight gain, FI, and FCR ●decreased diarrhea rate ●higher IgG, IgM, C-4, IL-2, T-AOC, SOD, GSH-Px in serum ●lower MDA in serum	[172]
Tannic acid	Type: weaned piglets (Duroc × Landrace × Yorkshine) dose: 500, 1000 and 1500 mg/kg source: commercial (Chinese gallnut) form: microencapsulated duration: 21-35 days	●no effects on ADG, FI, and FCR ●lower crypt depth with higher ratio of villus height to crypt depth of duodenum ●higher gene expression of solute carrier family 6, member 19 and solute carrier family 15, member 1 in ileum ●lower gene expression of solute carrier family 5, member 1 in jejunum ●lower maltase activities in ileum ●improved colonic bacterial community	[173]
Tannic acid	Type: piglets (Landrace × Yorkshine × Duroc) source: commercial (chestnut wood, 75% tannins) dose: 1000 mg/kg form: powder duration: 0-28 days	●no effects on ADG, FI, and FCR ●higher CAT, GSH-Px, IgM and lower MDA in serum ●improved trypsin, lipase and amylase activities ●higher villus height of jejunum ●higher propionic acid, butyric acid, and acetic acid concentrations in the colon ●lower diarrhea incidence	[6]
Tannins	Type: piglets (German Landrace × Pietrain) and *in-vitro* (cecal fermentation) dose: 1.13, 2.25, 4.5 g/kg; and 0.75, 1.5, 3, 6 g/L (*in vitro*) source: commercial (chestnut wood extract, 75% tannins) form: powder duration: 0-28 days	●lower total gas production, concentrations of ammonia and volatile fatty acids (*in vitro*) ●improved FCR ●decreased caecal concentrations of ammonia, iso-butyric, and iso-valeric acid ●higher number of lactobacilli in jejunum	[174]
Tannic acid	Type: fattening boars (Landrace × Large White) source: commercial extract (75% tannins) dose: 1%, 2% and 3% form: powder duration: 123-193 days	●higher villus height and mucosal thickness in duodenum ●decreased mitosis and apoptosis count in large intestine	[175]
Hydrolysable tannic acid	Type: Swiss Large White boars dose: 15 mg and 30 mg/kg source: commercial (chestnut) form: powder duration: 105-165 days	●no effects on FI, ADG and carcass traits ●improved feed efficacy ●reduced size of salivary and bulbourethral gland	[176]
Polyphenols (condensed tannins)	Type: MO25C-barrows (Moura × Landrace, and Large White) dose: 10% source: grape pomace form: powder duration: 21 days	●no effects on production of thiobarbituric acid reactive substances in the loin samples ●higher redness value of pork	[177]
Hydrolysable tannic acid	Type: male pigs (Landrace × Yorkshire × Duroc) source: commercial (albumin tannate, 500 g TA/kg) dose: 125, 250, 750 and 1000 mg/kg form: powder duration: 0-28 days	●reduced ADG but improve FCR ●lower faecal coliform count ●higher excretion of Fe in faeces ●lower total erythrocyte, hemoglobin, and hematocrit in plasma	[178]
Essential oil	Type: crossbred piglets (Duroc × Landrace × Large White) dose: 0.025% source: commercial (4.5% cinnamaldehyde and 13.5% thymol) form: powder duration: 0-28 days	●higher ADG, DM, CP, and energy retention ●higher villus height of jejunum ●lower *E. coli* and total anaerobes in rectum ●higher albumin, IgA, IgG, and T-AOC in plasma	[179]
Essential oil	Type: wined piglets (Duroc × Landrace × Yorkshire) dose: 50, 100, 150 g/t source: commercial (cinnamaldehyde and thymol) form: powder duration: 36-71 days	●higher FI, ADG, final body weight ●lower FCR ●decreased diarrhea ●lower *E. coli* in feces ●higher, IgA, IgG, C3, C4 in blood	[180]
Essential oil	Type: wined piglets (Duroc × Landrace × Yorkshire) dose: 30 mg/kg source: commercial (25% thymol and 25% carvacrol) form: powder duration: 0-28 days	●higher ADG, DM, CP, Ca, P, and GE digestibility ●higher number of *Lactobacilli* counts in feces ●higher villi height of duodenum ●higher trypsin and chymotrypsin activities	[181]
Essential oil	Type: male pigs (Duroc × Landrace × Large White) dose: 50, 100 and 200 ppm source: commercial (13.5% thymol and 4.5% cinnamaldehyde) form: silage duration: 53 days	●higher ADG and DM digestibility ●lower TC, TG in serum ●higher goblet cell and lactase activities in jejunum ●higher ratio of villus and crypt depth in ileum ●higher sucrose activities in duodenum ●higher expression of occluding and glucose transporter-2 gene in duodenum and ileum	[182]

## Conclusions

Although few studies noted that there were no any significant effects of dietary polyphenols on production performance and health status of farm animals but most of the studies found their efficacy to be used as potential feed additives. Also, irrespective of a large number of studies claiming health-promoting properties of various phenolic compounds, it should be mentioned that not all the phenolic compounds are necessarily beneficial, and their physiological effects depends on a range of factors. The action of phenolic was varied due to different sources, parts of plants, degree of maturity, dosages, extraction method, environmental or dietary patterns, metabolism, host species, duration of trail and bioavailability of compounds in some trials. Collectively this review highlighted to use phenolic compounds as a potential natural feed additive which has a role on the antioxidant, immunity, antimicrobial and overall production performance in poultry and swine. To get the advantages form those natural compounds, selected phenolic compound along with optimum level of supplementation should be considered base on specific animal species.

## Data Availability

Not applicable.

## Abbreviations

ADG: Average daily gain
FI: Feed intake
FCR: Feed conversion ratio
GST: Glutathione-S-transferase
GSH: Reduced glutathione
TBARS: Thiobarbituric acid reactive substances
CARB: Protein carbonyls
MDA: Malondialdehyde
CORT: Corticosterone
TC: Total cholesterol
LDL-C: Low-density lipoprotein cholesterol
HDL: High-density lipoprotein
TG: Triglyceride
ALT: Alanine aminotransferase
ALP: Alkaline phosphatase
AST: Aspartate aminotransferase
CRE: Creatinine
SFA: Saturated fatty acid
MUFA: Monounsaturated fatty acid
PUFA: Polyunsaturated fatty acid
IBV: Infectious bronchitis virus
IBDV: Infectious bursal disease virus
NDV: Newcastle disease virus
SRBC: Sheep red blood cell
C4: Complement 4
IL: Interleukin
ZO: Zonula occludens
TP: Total protein
P: Phosphorus
DM: Dry matter
CP: Crude protein
EE: Crude fat
GE: Gross energy
